# Sex, Sex Hormones and Inflammatory Burden and Cardiovascular Risk in Patients with Spondyloarthritis

**DOI:** 10.3390/biom16091345

**Published:** 2026-09-16

**Authors:** Isaac T. Cheng, Huan Meng, Ho So, Lai Shan Tam

**Affiliations:** Department of Medicine and Therapeutics, The Chinese University of Hong Kong, Shatin, Hong Kong, China; ichength@cuhk.edu.hk (I.T.C.); huanmeng@cuhk.edu.hk (H.M.); hoso@cuhk.edu.hk (H.S.)

**Keywords:** sex, sex hormone, spondyloarthritis, psoriatic arthritis, inflammatory burden, cardiovascular risk

## Abstract

Spondyloarthritis encompasses a spectrum of inflammatory diseases, including axial spondyloarthritis and peripheral forms such as psoriatic arthritis. Chronic inflammation is closely linked to an increased risk of cardiovascular (CV) disease in these patients. Beyond inflammatory burden, biological sex and sex hormones may modify both inflammatory pathways and CV risk. Although the mechanisms underlying sex differences in spondyloarthritis remain incompletely understood, emerging evidence suggests clinically relevant differences in disease manifestations, inflammatory activity, and CV outcomes between males and females. This review summarizes current evidence on the interplay between sex, sex hormones, inflammatory burden, and CV risk in patients with spondyloarthritis, and highlights potential implications for risk stratification and future research.

## 1. Introduction

Spondyloarthritis (SpA) comprises a group of chronic inflammatory diseases affecting the axial and peripheral joints, with axial spondyloarthritis (axSpA) and psoriatic arthritis (PsA) representing two major clinical entities within the SpA spectrum [1]. Patients with SpA have an increased risk of cardiovascular (CV) disease, as demonstrated across multiple meta-analyses [2,3]. This excess risk may be attributable not only to traditional CV risk factors including sex, but also to the cumulative inflammatory burden associated with SpA [4,5].

Clinical trials were usually underpowered to explore sex differences, especially in axSpA as a male-dominant disease [6]. Nevertheless, in recent years, sex differences in disease phenotype, clinical presentation, inflammatory burden, and treatment outcomes have been increasingly recognized [7]. As sex may also influence CV risk, an integrated review of the role of sex in shaping inflammatory burden and CV risk in SpA is warranted.

Therefore, this review aims to summarize current evidence on how sex modifies the expression, assessment, accumulation, and treatment responsiveness of inflammatory burden across the SpA spectrum, and how these processes may shape CV risk trajectories. Recognizing sex as a clinical modifier may support more precise risk stratification and sex-aware management in SpA.

There is currently no universally accepted definition of inflammatory burden in SpA. In this review, the term is used broadly to describe the multidimensional impact of inflammatory disease, encompassing: (1) clinical manifestations and symptom burden, (2) objective inflammatory activity assessed by acute-phase reactants and imaging, (3) treatment-related outcomes, and (4) cumulative structural consequences of disease. These domains are related but not interchangeable and may exhibit discordant patterns, particularly between sexes. It is also recognized that symptoms and treatment responsiveness may be influenced by comorbidities such as fibromyalgia. Sex refers to biological sex commonly categorized as male or female and defined by biological attributes including sex chromosomes, reproductive anatomy, and sex hormone profiles. Sex may influence disease pathophysiology, clinical manifestations, inflammatory burden, treatment response, and comorbidities [7,8]. This differs from gender, which refers to socially constructed norms and roles that may affect disease perception, health-seeking behavior, decision-making, and the use of healthcare services [7,8]. Although sex and gender interact in complex ways, this review focuses on the role of biological sex in influencing inflammatory burden and CV risk in patients with SpA.

This narrative review was based on a literature search conducted in PubMed. Searches were performed using combinations of keywords including “spondyloarthritis”, “axial spondyloarthritis”, “ankylosing spondylitis”, “psoriatic arthritis”, “sex differences”, “inflammatory burden”, “cardiovascular risk”, “major adverse cardiovascular events (MACE)”, and “atherosclerosis”. Original studies, observational cohorts, clinical trials, and relevant review articles addressing sex differences in inflammatory burden and cardiovascular outcomes in SpA were considered. Additional relevant publications were identified through manual screening of reference lists. Articles were selected according to their relevance to the scope of this review. The search included articles published up to June 2026 and was limited to English-language publications.

## 2. Sex Difference in Inflammatory Burden

### 2.1. axSpA

#### 2.1.1. Clinical Manifestations and Symptom Burden

Although male and female patients share the same diagnosis of axSpA, the clinical manifestations appear to differ by sex. In terms of clinical symptoms, female patients have been reported to experience more widespread pain, including pain in the upper back and cervical spine, beyond classic inflammatory low back pain [6,9]. Overall, perceived pain levels were also higher in female than in male patients [9]. Regarding enthesitis, several studies have consistently reported higher Maastricht Ankylosing Spondylitis Enthesitis Score (MASES) values in female patients [9,10,11].

These symptoms are not confined to the axial compartment. Female patients have been reported to have higher peripheral tender and swollen joint counts [9,10,11]. They also appear to have a higher prevalence of extra-articular manifestations, including inflammatory bowel disease (IBD) [6,12,13] and psoriasis [13,14].

Radiographically, fewer female patients are classified as having radiographic axSpA (r-axSpA) [9,11,15], whereas the prevalence of non-radiographic axSpA (nr-axSpA) appears to be similar between sexes [6].

The differential expression of inflammatory burden, particularly widespread pain in female patients, may contribute to longer diagnostic delay, partly because axSpA may initially be misdiagnosed as fibromyalgia [16]. In a meta-analysis, the average diagnostic delay was 8.8 years in female patients compared with 6.5 years in male patients [17]. Female patients also reported a longer duration of inflammatory back pain (IBP) than male patients, even at an early disease stage [11]. Diagnostic delay may result in persistent untreated inflammation and could promote the development of nociplastic pain mechanisms or central sensitization [18], which may amplify pain perception independently of objective inflammatory activity. These findings suggest that sex may influence the clinical expression of inflammatory burden, particularly through differences in pain perception, extra-articular involvement, and diagnostic trajectories.

#### 2.1.2. Composite Disease Activity Assessment

The magnitude of inflammatory burden in axSpA is commonly assessed using composite disease activity indices, including the Bath Ankylosing Spondylitis Disease Activity Index (BASDAI) and the Axial Spondyloarthritis Disease Activity Score (ASDAS). Across studies, female patients have consistently reported higher disease activity than male patients [9,10,15,19,20].

#### 2.1.3. Objective Inflammatory Activity

In a 6-year study of early axSpA, female patients had higher ASDAS and patient global assessment scores than male patients at all time points, despite having lower C-reactive protein (CRP) levels over time [11]. Similarly, several studies have reported less MRI-detected inflammation in the sacroiliac joints and/or spine among female patients [9,11,21].

This apparent discordance suggests that sex differences in disease activity may not be explained by objective inflammatory markers alone. Patient-reported pain, global assessment, fatigue, and other symptom-related domains may contribute substantially to higher composite disease activity scores in female patients, influencing the overall assessment of inflammatory burden despite objective findings of lower CRP levels and less inflammatory changes on MRI.

#### 2.1.4. Treatment-Related Outcomes

Upon appropriate treatment, objective inflammatory markers generally improve over time. However, treatment exposure appears to differ between sexes. In a US registry, female patients received more conventional synthetic disease-modifying antirheumatic drugs (csDMARDs) and oral glucocorticoids than male patients prior to initiation of biologic DMARDs (bDMARDs) [10]. They also received more non-steroidal anti-inflammatory drugs (NSAIDs). In an insurance claims–based study, female patients were more likely to continue NSAIDs after initiation of bDMARDs [7]. One possible explanation is greater residual disease activity in female patients despite exposure to different therapeutic agents. Indeed, a Spanish registry reported that female patients were less likely than male patients to achieve BASDAI50 after first-line anti–tumor necrosis factor (anti-TNF) treatment [19]. Similarly, a French study reported an odds ratio (OR) of 3.23 for failure to achieve BASDAI50 in female patients compared with male patients [22]. With regard to interleukin-17 inhibitor (IL-17i) treatment, an early ASAS40 response was observed in male patients, whereas it was less evident in female patients [23]. A recent meta-analysis similarly reported that male patients had a better response to biologic or targeted synthetic DMARDs (b/tsDMARDs), with an OR of 1.89 for achieving ASAS40 compared with female patients [24].

#### 2.1.5. Structural Consequences and Disease Progression

Despite more favorable responses to therapy in male patients, structural progression appears to be greater in males than in females. In the DESIR cohort, female patients maintained higher ASDAS scores throughout 6 years of follow-up, yet CRP levels were comparable between sexes. Notably, radiographic damage accumulated more rapidly in male patients [11]. This apparent dissociation between patient-reported disease activity assessments, objective inflammatory markers, and structural progression suggests that the biological consequences of inflammation may differ by sex. Whereas female patients may experience a greater symptomatic burden and lower treatment responsiveness, male patients appear more susceptible to inflammation-driven structural damage. Consequently, inflammatory burden should not be interpreted solely through composite disease activity measures, as its long-term structural consequences may vary substantially between sexes.

### 2.2. PsA

#### 2.2.1. Clinical Manifestations and Symptom Burden

PsA is a heterogeneous disease, and its clinical expression appears to differ between sexes. Female patients more commonly present with a polyarticular pattern, whereas male patients are more likely to present with oligoarticular disease or isolated distal interphalangeal joint involvement [25,26]. Axial involvement has also been reported more frequently in male patients [26].

Consistently, two meta-analyses of observational studies and randomized controlled trials reported higher pain scores, tender joint counts, and enthesitis in female patients [27,28]. In contrast, male patients were more likely to have dactylitis [27]. The number of swollen joints appears to be less consistently dominated by either sex [27,28]. Regarding the skin domain, male patients have consistently been reported to have more severe psoriasis [27,28].

For extra-articular manifestations, a population-based study reported similar prevalence of uveitis and IBD between male and female patients with PsA [29].

Although diagnostic delay appears to be longer in female patients with axSpA, this pattern may not apply equally to PsA. In a Chinese study, diagnostic delay was comparable between sexes, with a median (IQR) delay of 19.5 (5.5, 32.5) months in male patients and 18 (5, 27) months in female patients [30].

Together, these findings suggest that sex may influence the clinical expression of PsA in a domain-specific manner. Female patients tend to show a more symptom- and peripheral joint–dominant phenotype, whereas male patients more often show axial involvement, dactylitis and more severe skin disease.

#### 2.2.2. Composite Disease Activity Assessment

Female patients generally have higher composite disease activity scores across study time points [28]. In clinical trials, male patients were more likely to achieve minimal disease activity (MDA), regardless of the class of therapeutic agent received [27], and similar observations have been reported in observational studies [28]. These findings suggest that female patients may experience a higher clinical disease burden when assessed using composite disease activity measures.

#### 2.2.3. Objective Inflammatory Activity

However, composite indices may not fully distinguish between inflammatory activity and symptom-related domains such as pain, tender joints, fatigue, and patient global assessment. Objective imaging findings provide a more nuanced picture. In an Israeli study, DAPSA scores were comparable between sexes, but ultrasound-detected inflammatory lesions were more frequent in male patients than in female patients [31]. Ultrasound total scores, greyscale scores, and power Doppler scores for enthesitis were also higher in male patients [31]. Notably, these imaging differences were not reflected by clinical enthesitis scores based on palpation, highlighting a potential discordance between clinical assessment and objective inflammatory signals.

Therefore, sex differences in the magnitude of inflammatory burden in PsA should be interpreted according to the domain being measured. Female patients may show higher clinical or composite disease activity, whereas male patients may demonstrate greater objective inflammatory signals on imaging in some settings. This suggests that sex may influence the relationship between symptoms, composite indices, and objective inflammation, rather than uniformly increasing inflammatory burden in one sex.

#### 2.2.4. Treatment-Related Outcomes

The accumulation of inflammatory burden over time may be influenced by treatment response, persistence, and structural progression, all of which appear to differ by sex. In an Italian study of prescription patterns, male patients were more likely to receive csDMARD monotherapy, whereas female patients were more likely to receive bDMARDs [32]. Female patients were also more likely to experience at least one switch or swap of bDMARD therapy [32]. These findings may reflect sex-related differences in treatment response, tolerability, patient preference, perceived effectiveness, or prescribing behavior.

Real-world registry data further suggest sex differences in treatment persistence. The European Spondyloarthritis Research Collaboration Network registries reported lower retention of first-line TNFi treatment in female patients receiving b/tsDMARDs for PsA [33]. A Japanese study similarly reported lower biologic retention in female patients across different biologic classes [34]. However, data from a French insurance claims database suggested that persistence was similar between sexes among IL-12/23i, IL-23i, and JAKi users, while persistence was lower in female patients receiving TNFi and IL-17i [35]. Taken together, these findings suggest heterogeneous and treatment class–specific sex differences in persistence, rather than a uniform difference across all therapies.

Importantly, treatment retention should not be interpreted as a direct measure of efficacy alone, as it may reflect a combination of effectiveness, safety, tolerability, patient preference, comorbidities, and healthcare-related factors. Nevertheless, trial data also suggest sex differences in treatment response. In an earlier meta-analysis, ACR response rates were higher in male patients treated with TNFi, IL-12/23i, IL-17i, and IL-23i, whereas responses were similar between sexes among JAKi users [27].

#### 2.2.5. Structural Consequences and Disease Progression

Despite evidence suggesting lower clinical response rates and/or treatment persistence among female patients, radiographic progression in both axial and peripheral domains appears to be more severe in male patients [25]. Female patients were more likely to have non-erosive disease or milder structural damage than male patients [25]. This discordance suggests that sex may differentially shape symptomatic disease burden, treatment response, persistence, and structural damage in PsA, rather than exerting a uniform effect across all disease domains.

Integrating the findings in Section 2, it is demonstrated that different domains of inflammatory burden may not align. Female patients generally report higher symptom burden and disease activity scores, whereas male patients more frequently exhibit higher objective inflammatory activity and greater structural progression. Therefore, sex differences should be interpreted according to the specific domain being evaluated rather than assuming a uniform pattern across all measures. Table 1 summarizes the above according to various domains.

## 3. Sex Difference in CV Risk

The influence of sex on CV risk in SpA may be indirect rather than direct. Sex may influence inflammatory burden as discussed in Section 2, and inflammatory burden may, in turn, contribute to vascular injury, atherosclerosis and subsequent CV events. Accordingly, CV risk in SpA can be considered across three sequential tiers, each of which may show sex-specific patterns.

The first tier comprises traditional, modifiable CV risk factors, including hypertension, dyslipidemia, diabetes mellitus, smoking and physical inactivity. These risks may also be captured by conventional risk prediction models such as the Systematic Coronary Risk Evaluation (SCORE) [36] and the Framingham risk score [37]. The second tier is subclinical atherosclerosis, which could be assessed using markers such as carotid intima-media thickness (IMT) and the presence of carotid or coronary plaque [38]. These measures independently predict future CV events. The third tier comprises hard event outcomes, including major adverse cardiovascular events (MACE), namely myocardial infarction, stroke and CV death.

### 3.1. axSpA

#### 3.1.1. Distribution of Traditional CV Risk Factors

Available evidence suggests that the distribution of traditional CV risk factors in axSpA differs by sex. Reviews consistently report a higher prevalence of smoking among male patients, whereas female patients tend to have higher BMI and greater adiposity [6,39,40]. The Spanish multicenter AtheSpAin cohort—the largest dedicated CV study in axSpA—demonstrated that male patients had a higher prevalence of smoking, hypertension, and dyslipidemia, together with a more atherogenic lipid profile characterized by lower HDL-cholesterol, higher triglyceride levels, a higher atherogenic index, and higher blood pressure. In contrast, female patients exhibited greater central adiposity and a higher triglyceride–glucose index. Meanwhile, the prevalence of diabetes was comparable between sexes [41]. Collectively, these findings suggest distinct cardiometabolic profiles in axSpA, with males exhibiting a predominantly atherogenic risk factor pattern and females exhibiting a more central, metabolically adverse pattern of adiposity.

#### 3.1.2. Subclinical Atherosclerosis and CV Events

Although accelerated atherosclerosis is well documented in axSpA [5], evidence regarding sex differences in subclinical vascular disease remained limited. In the AtheSpAin cohort, female patients had a lower carotid IMT than male patients after adjustment for traditional CV risk factors [41]. However, among patients classified as having high or very high CV risk according to SCORE, carotid plaques were more frequently observed in female patients despite having a lower traditional CV risk profile. Notably, the female group had higher disease activity and greater functional impairment, suggesting that inflammatory burden may alter the relationship between sex and subclinical atherosclerosis [41]. By contrast, a smaller cross-sectional study found no association between sex and carotid IMT in either axSpA or healthy controls [42]. Taken together, current evidence is insufficient to establish a consistent sex difference in subclinical atherosclerosis in axSpA.

Data on clinical vascular outcomes are similarly heterogeneous. A Taiwanese nationwide cohort reported comparable risks of acute coronary syndrome in male and female patients with axSpA [43]. For cerebrovascular disease, one Swedish registry found that female patients had a higher risk of stroke compared with the general population, whereas no significant increase was observed among male patients. Findings regarding thromboembolic events are conflicting. The same Swedish study reported a higher risk of thromboembolic events in male patients [44], whereas a second Swedish study found venous thromboembolism to be more common among female patients in axSpA [45]. The discrepancies may be explained by differences in follow-up design, inclusion criteria and, notably, stroke-endpoint definition (whether TIA and unspecified stroke were included). With regard to mortality, Canadian population-based data reported a significantly increased risk of vascular mortality in male patients with axSpA (HR 1.46, 95% CI 1.13–1.87), whereas the association did not reach statistical significance in female patients (HR 1.24, 95% CI 0.92–1.67) [46]. Overall, findings for subclinical atherosclerosis, CV events, and CV mortality remain inconsistent, and current data do not support a clear predominance of CV risk in either sex. Interpretation is further limited by the male predominance of many axSpA cohorts and the scarcity of dedicated sex-stratified analyses.

### 3.2. PsA

#### 3.2.1. Distribution of Traditional CV Risk Factors

Similar to patients with axSpA, male and female PsA patients had differential traditional CV risk profiles. In a Swedish cohort, female patients with PsA had a higher prevalence of multiple CV risk factors than age-matched controls, including obesity, hypertension, diabetes, and smoking. In contrast, among male patients, only obesity and hypertension were more prevalent than in controls [47]. Consistent with this, a cross-sectional study of 102 patients found that the prevalence of metabolic syndrome was significantly higher in female patients than in male patients with PsA [48]. A comprehensive review reached the same conclusion, noting that among the traditional CV risk factors, the prevalence of metabolic syndrome, diabetes, and hypertension has been reported to be higher in female patients with PsA [25]. Taken together, these observations point toward a female-predominant burden of conventional CV risk factors in PsA.

These traditional CV risk factors should not be viewed in isolation, as they frequently coexist as cardiometabolic multimorbidity. In PsA, a systematic review and meta-analysis confirmed a high pooled prevalence of interrelated cardiometabolic conditions—obesity, hypertension, diabetes, and metabolic syndrome—rather than isolated risk factors [49]. As several of these are more prevalent in female patients, this multimorbidity burden may itself differ by sex. Although derived from rheumatoid arthritis and not directly transferable to PsA, a real-world study found that cardiometabolic multimorbidity was associated with a more severe disease phenotype [50]. This suggests that the clustering of multiple comorbidities, rather than any single factor alone, may contribute to disease severity and cardiovascular risk. Whether such clustering differs by sex in PsA warrants further investigation.

#### 3.2.2. Subclinical Atherosclerosis and CV Events

Interestingly, although females appeared to have a higher prevalence of traditional CV risk factors, current evidence does not consistently demonstrate a higher burden of subclinical atherosclerosis or adverse CV outcomes compared with males. From a Hong Kong cohort, male sex was associated with higher odds of subclinical carotid atherosclerosis when compared with female (OR 3.63, 95% CI 1.33–9.91) [51]. Another Spanish multi-territory study also reported that female sex was independently associated with a lower likelihood of carotid plaque (OR 0.48, 95% CI 0.24–0.98) and, more strikingly, of femoral plaque (OR 0.14, 95% CI 0.05–0.35) and of multisite disease (OR 0.32, 95% CI 0.15–0.69) after multivariable adjustment [52]. Another Hong Kong study reported data on coronary atherosclerosis detected by coronary computed tomography angiography, which linked male sex to the presence of vulnerable plaque [53]. Meanwhile, other studies have reported similar carotid IMT or prevalence of carotid plaque [54] between sexes.

Sex differences in clinical CV outcomes generally mirror the pattern observed in subclinical atherosclerosis. A Japanese survey reported a higher prevalence of CV disease (CVD) (5.0% vs. 2.5%) among male than female patients with PsA [55]. Similarly, a UK nationwide study reported a higher CVD rate in male than female patients with PsA (4.3 vs. 2.3%) [56]. Regarding mortality, a UK study of severe PsA found that mortality from circulatory disease, particularly coronary heart disease, was significantly elevated in male patients relative to the general population but not in female patients [57]. Overall, the available evidence suggests that although female patients with PsA may carry a greater burden of traditional CV risk factors, male patients appear more likely to develop subclinical atherosclerosis, experience CV events, and die from CV causes. The overall findings were summarized in Table 2.

## 4. Potential Biological Mechanisms Underlying Sex Differences?

### 4.1. Sex-Specific Genetic and Epigenetic Mechanisms

AxSpA has a strong genetic predisposition linked to human leukocyte antigen B27 (HLA-B27) [40]. HLA-B27 positivity is also associated with radiographic axSpA and more severe structural damage [58]. Numerous studies have reported a lower prevalence of HLA-B27 in female patients [9,11,19,59]. This difference may partly contribute to sex-related discrepancies between the clinical expression of inflammatory burden and the accumulation of inflammatory burden as reflected by radiographic damage.

Sex-specific genetic and epigenetic regulation may contribute to differences in disease susceptibility and phenotype. One study reported increased hypermethylation of the *IL22* gene in sperm cells from patients with PsA, but not in patients with psoriasis alone [60], suggesting a potential germline epigenetic signal related to PsA susceptibility. Sex-specific genetic associations have also been described. The ANKH gene encodes a protein involved in pathological ankylosis in radiographic axSpA. In one study, different *ANKH* loci were associated with ankylosing spondylitis (AS) in male and female patients: the haplotype combination of rs26307 and rs27356 was significantly associated with AS in male patients, whereas the haplotype combination of rs28006 and rs25957 was significantly associated with AS in female patients. A sex interaction was also observed for rs26307, indicating that the strength of genetic association may differ by sex [61]. Similar to axSpA, PsA has a strong genetic component, with the most consistent genetic contribution arising from the major histocompatibility complex (MHC) class I region, alongside non-HLA genes encoding proteins in the TNF and IL-23/IL-17 pathways [62]. Among discordant parental pairs, a significantly larger proportion of probands reported an affected father than an affected mother (57% vs. 43%; OR 1.3, 95% CI 1.1–1.6, *p* = 0.003), a paternal transmission bias that also held within PsA probands (*p* = 0.007) [63]. This pattern is consistent with genomic imprinting—an epigenetic phenomenon in which genes are expressed in a parent-of-origin–specific manner—and an imprinting-based linkage analysis mapped a PsA susceptibility gene to chromosome 16q [64], reaching significance only when the analysis was conditioned on paternal transmission [65]. Beyond imprinting, sex differences in inflammatory cytokine production have also been linked to X-chromosome gene dosage: the X chromosome is enriched for immune-related genes, some of which escape X-inactivation and are expressed biallelically at higher levels in female patients, thereby altering autoimmune susceptibility [66,67]. Consistent with this, one study found higher levels of IL-1β, IL-6, IL-8, TNF and IFNα in male patients than in female patients after immune-cell stimulation [68].

Taken together, these findings suggest that biological sex may influence disease susceptibility and trajectory from an early stage through genetic and epigenetic mechanisms. Such inherent differences may, in turn, shape downstream inflammatory burden and potentially affect long-term CV risk.

### 4.2. Sex Hormones

Sex hormones may influence immune regulation and thereby shape the underlying inflammation in SpA. Testosterone has been reported to exert an anti-inflammatory effect by promoting IL-10, which in turn suppresses TNF and other pro-inflammatory pathways [69,70]. In addition, testosterone may increase pain threshold [71]. These mechanisms may partly explain the observation that female patients often report higher subjective pain levels despite lower levels of objective markers like CRP or inflammatory signal on ultrasound in some studies.

On the contrary, estrogen may exert pro-inflammatory effects under certain conditions by enhancing both cell-mediated and humoral responses. It promotes the production of pro-inflammatory cytokines such as IL-1, IL-6 and TNF [69,70]. The fluctuation in pain intensity across the menstrual cycle in females, compared with the relatively stable pain profile in males, might further support the concept of a role for sex hormones in modulating pain perception and inflammatory response [72,73].

Nevertheless, beyond these proposed biological mechanisms, current evidence remains inconsistent. Some studies have reported higher testosterone levels in male r-axSpA patients than in healthy controls [74], whereas others have demonstrated an inverse association between testosterone levels and disease activity in PsA [75]. In contrast, several studies have found no significant differences in sex hormone levels between patients with SpA and healthy individuals [76,77].

While these immunomodulatory effects provide a biologically plausible explanation for sex differences in inflammatory burden, direct evidence linking circulating sex hormone levels to disease activity, structural progression, or CV outcomes in SpA remains limited. Current clinical studies have yielded inconsistent findings, and hormone concentrations alone may not fully capture the complex interaction between sex hormones, immune pathways, and disease manifestations. In addition, an important limitation is that current studies did not account for menopausal status, reproductive stage, or hormonal therapies. As menopause may influence inflammation, adiposity, and cardiovascular risk, future studies are needed to clarify its contribution to sex-related differences observed in SpA.

Future research integrating hormonal profiling with clinical, imaging, and immunological assessments may help establish a more robust mechanistic framework for understanding how sex hormones influence inflammatory burden and disease outcomes in SpA.

### 4.3. Sex-Specific Endocrine Response

Adipokines are a group of endocrine mediators secreted by adipose tissue that may modulate immune responses and contribute to the pathogenesis of SpA. Adipose tissue and inflammation are closely interconnected. One cross-sectional study reported that greater adiposity may promote inflammation, whereas chronic TNF-driven inflammation may conversely induce anorexia, increase resting energy expenditure, cause loss of muscle mass, and down-regulate anabolic hormones and growth factors—changes that may manifest as cachexia [78]. This bidirectional relationship suggests that adipose tissue may act not only as a consequence of inflammatory burden but also as a driver of it.

Importantly, the expression of several adipokines differs between males and females. Leptin is a pro-inflammatory adipokine that, beyond its primary role in regulating energy balance and metabolism, promotes T-cell and B-cell proliferation, activates monocytes and macrophages, and stimulates the production of pro-inflammatory cytokines such as IL-1, IL-6, and TNF [79]. Leptin may also enhance the activation of dendritic cells and natural killer cells, thereby amplifying inflammatory responses [79]. Notably, circulating leptin levels have consistently been reported to be higher in females than in males, independent of body mass index [80,81,82]. Resistin is another adipokine with predominantly pro-inflammatory properties. It promotes cytokine production by monocytes through increasing endothelial permeability and inducing adhesion molecules, thereby facilitating inflammatory cell recruitment. Similar to leptin, higher resistin level have been reported in female r-axSpA patients [81]. Compared with healthy controls, patients with PsA exhibit increased leptin and resistin together with reduced adiponectin, changes that correlate with disease activity, structural damage, and subclinical CV abnormalities [83,84,85,86]. Whether these adipokine alterations differ by sex in PsA specifically has, however, rarely been examined; most PsA adipokine studies have not been sex-disaggregated.

In contrast, omentin is generally considered an anti-inflammatory adipokine. It may exert its effects through activating M2 macrophages and suppressing the production of pro-inflammatory cytokines. Higher circulating omentin levels have also been observed in female patients with radiographic axSpA [87].

Beyond differences in adipokine concentrations, sex-specific body composition may further influence adipokine-related immune regulation. In general, males tend to have a greater proportion of intra-abdominal visceral adipose tissue, whereas females exhibit relatively greater subcutaneous fat deposition [88]. These differences in fat distribution may contribute to variations in adipokine secretion profiles, although the effects of some adipokines appear to be independent of body weight. The overall relationship between adipokines and inflammatory burden is likely to be complex and multifactorial.

Sex-specific differences in adipokine profiles may represent another biological pathway through which sex modifies inflammatory burden. However, current evidence is largely observational, and the extent to which adipokines mediate the clinical differences observed between male and female patients with SpA remains uncertain.

### 4.4. Inflammation as a Mediator of CV Risk

While sex-specific genetic, hormonal and endocrine factors may influence inflammatory burden, inflammation itself is increasingly recognized as a key driver of CV risk in SpA. Importantly, emerging evidence suggests that the CV consequences of inflammation may differ between males and females, potentially contributing to the sex-specific CV phenotypes observed in axSpA and PsA.

In the AtheSpAin cohort, the relationship between inflammation and cardiometabolic risk factors differed by sex. In male patients, acute-phase reactants were primarily associated with HDL-cholesterol levels. In female patients, however, inflammatory markers were associated with triglycerides, insulin resistance parameters, and glucose metabolism. Notably, an independent association between inflammation and glucose metabolism was observed only in female patients [89]. Findings on hypertension are broadly concordant: a Hong Kong longitudinal cohort found ESR to be independently associated with incident hypertension in axSpA [90], and in the AtheSpAin cohort elevated baseline acute-phase reactants predicted hypertension years later in both sexes [89]. For lipids specifically, a 2023 systematic review and meta-analysis examined individual serum lipids in axSpA. It found that a lower mean HDL-cholesterol was the only consistent lipid alteration, with no significant change in mean triglycerides [91]. This pattern may itself reflect the male predominance of axSpA cohorts. Indeed, the AtheSpAin data suggest that the link between inflammation and HDL-cholesterol is largely confined to male patients [89].

The impact of inflammation extends beyond traditional CV risk factors to vascular pathology itself. While subclinical atherosclerosis is a surrogate marker of CV risk, plaque burden and vascular inflammation may improve with effective control of disease activity. However, the vascular response to treatment may also differ by sex. Reductions in plaque progression and vascular inflammation during TNFi therapy have been reported predominantly in male patients [92], whereas the response to cytokine-targeted therapies in female patients remains uncertain.

Regarding MACE and CV mortality, a 2023 systematic review concluded that persistently active disease is consistently associated with MACE and CV mortality in axSpA, whereas effective suppression of inflammation with biologic therapy appears to attenuate this excess risk [5]. However, these outcome data are not sex-stratified, and whether a given inflammatory burden confers a disproportionate CV risk in female patients remains an open question requiring prospective investigation.

**Figure 1:**
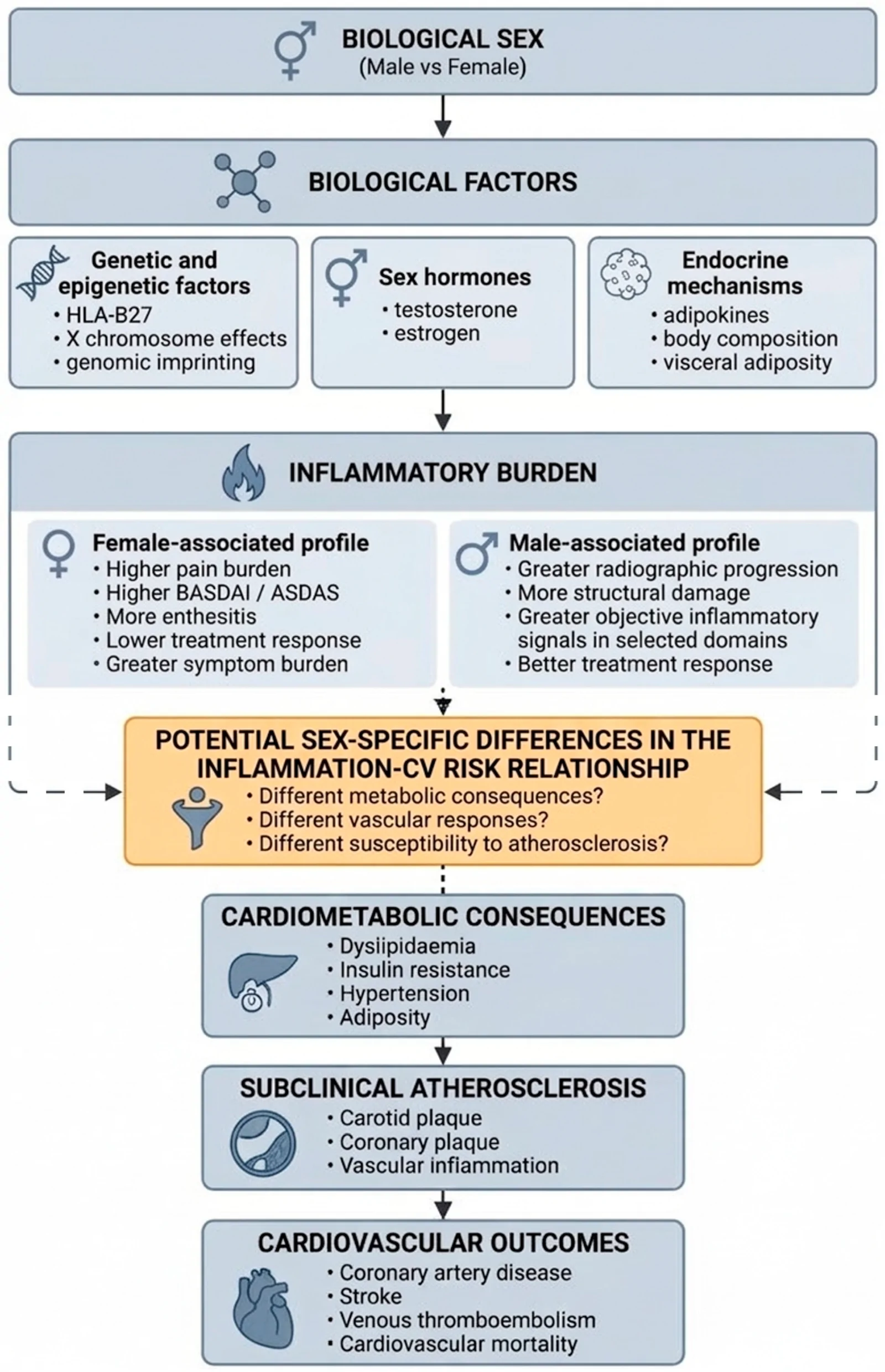
Conceptual framework illustrating the role of biological sex in shaping inflammatory burden and cardiovascular risk in spondyloarthritis. Sex-specific genetic, hormonal, and endocrine factors may influence inflammatory burden, while both biological sex and inflammation contribute to cardiometabolic dysfunction and cardiovascular outcomes.

Collectively, these findings support that inflammation may be an important contributor to the relationship between biological sex and CV risk in SpA. Table 3 summarized the current findings. However, most outcome studies have not been adequately sex-stratified, and whether a comparable inflammatory burden confers different CV consequences in male and female patients remains unresolved. Addressing this knowledge gap will require prospective studies specifically designed to evaluate sex-specific CV outcomes. A conceptual framework illustrating the role of biological sex in shaping inflammatory burden and CV risk in SpA is illustrated in Figure 1.

## 5. Implication and Future Research

Recognizing sex as an important clinical factor influencing inflammatory burden and a mediator of CV risk may support a more sex-aware approach in disease management of SpA [7]. While the clinical manifestations, inflammatory burden, disease trajectory, treatment response and related comorbidities could vary by sex, more personalized management strategies that incorporate sex as a clinical modifier should be explored to optimize treatment and risk management in patients with SpA. Whether sex-specific management strategies improve clinical and CV outcomes compared with conventional approaches remains to be determined.

Although sex differences in inflammatory burden and CV risk have been consistently reported, the mechanistic pathways linking these observations remain incompletely understood. Genetic predisposition, sex hormones, adipokine biology, and differential inflammatory responses may all contribute, but direct evidence supporting these pathways remains limited. Future studies with comprehensive longitudinal hormonal data, along with changes in disease activity across sex, may provide further insights beyond mechanistic hypotheses.

In addition, there are limited data on sex-specific traditional CV risk profile, subclinical atherosclerosis, MACE and related mortality to draw a solid conclusion. Large-scale, multinational, prospective cohorts with dedicated sex-stratified analyses are needed to better define sex differences in traditional CV risk factors, subclinical atherosclerosis, CV events, and CV mortality in SpA. In particular, whether inflammation confers differential CV risk in male and female patients remains an important unanswered question. A summary of current evidence, major limitation and future research priority investigating sex differences in inflammatory burden and CV risk in SpA has been enlisted in Table 4. Several limitations of the current review should be acknowledged. Although this review focuses on male and female biological sex, sex minorities remain under-represented in clinical studies [7]. The interplay among sex, gender, and hormones is likely to be even more complex. Future studies incorporating transgender and gender-diverse populations may provide unique insights into the relative contributions of biological sex, sex hormones, and gender-related factors to disease expression and CV risk.

## 6. Conclusions

Current evidence suggests that biological sex influences both inflammatory burden and CV risk in SpA, although the relationship between these two domains remains incompletely understood. Sex differences have been observed in disease expression, traditional CV risk factors, subclinical atherosclerosis, and CV outcomes across axSpA and PsA, but the magnitude and direction of these differences vary between disease subtypes, especially in axSpA. Emerging evidence indicates that genetic, hormonal, and endocrine mechanisms may contribute to sex-specific inflammatory phenotypes, while inflammation itself may represent an important link between sex and CV risk. However, direct evidence demonstrating that inflammatory burden mediates sex differences in CV outcomes remains limited. Future studies integrating clinical, imaging, biochemical, hormonal, and CV outcome data are needed to clarify these relationships and support the development of more personalized approaches to CV risk assessment and management in SpA.

## Figures and Tables

**Table 1:** Sex-related differences in inflammatory burden in terms of clinical manifestations and symptom burden, objective inflammatory activity, composite disease activity assessment, treatment-related outcomes, and structural consequences and disease progression across axial spondyloarthritis and psoriatic arthritis.

	axSpA		PsA	
	Female	Male	Female	Male
**Clinical manifestations and symptom burden**				
Symptoms	↑ Widespread pain & overall pain level [6,9]		↑ Pain score & tender joint count [27,28]	
	↑ Enthesitis [9,10,11]		↑ Enthesitis [27,28]	
Disease phenotype		↑r-axSpA [9,11,15]		↑ Axial involvement [26]
	↑ Peripheral arthritis [9,10,11]		↑ Polyarticular pattern [25,26]	↑ Oligoarticular/ isolated DIP involvement [25,26]
				↑ Dactylitis [27]
Extra-articular manifestations	↑ IBD [6,12,13], PsO [13,14]			↑ PsO severity [27,28]
Diagnostic delay	↑ Delay [17]			
**Composite disease activity assessment & objective inflammatory activity**				
Composite disease activity score/state	↑ BASDAI/ASDAS [9,10,15,19,20]		↑ Composite disease activity score [28]	↑ MDA achievement [27,28]
Imaging		↑ Inflammation in SIJ/spine on MRI [9,11,21]		↑ Inflammatory signal on ultrasound^ [31]
**Treatment-related outcomes**				
Treatment exposure	↑ NSAIDs [7]/ csDMARDs [10]/oral glucocorticoids use [10]		↑ bDMARDs use [32] ↑ Switching/swapping of bDMARDs [32,33,34]	↑ csDMARD monotherapy [32]
Treatment response		↑ Response to b/tsDMARDs [22,23,24]		↑ ACR response to bDMARDs [27]
**Structural consequences and disease progression**				
Radiographic damage		↑ Damage [11]		↑ Damage [25]

Findings represent overall trends reported across multiple observational studies, systematic reviews, and meta-analyses. Where conflicting evidence exists, the predominant direction of association is shown. ↑ indicate a higher value compared with patients of the opposite sex within the same disease. The ultrasound findings denoted by ^ are based on a single study. The details are illustrated in Section 2. IBD—inflammatory bowel disease; PsO—psoriasis; r-axSpA—radiographic axial spondyloarthritis; BASDAI—Bath Ankylosing Spondylitis Disease Activity Index; ASDAS—Axial Spondyloarthritis Disease Activity Score; SIJ—sacroiliac joint; MRI—magnetic resonance imaging; csDMARDs—conventional synthetic disease-modifying antirheumatic drugs; b/tsDMARDs—biologic/targeted synthetic DMARDs; DIP—distal interphalangeal joint; MDA—minimal disease activity.

**Table 2:** Sex-related differences in CV risk across the three tiers of risk in axial spondyloarthritis and psoriatic arthritis.

	axSpA		PsA	
	Female	Male	Female	Male
**Traditional CV risk factor profile**				
Lipids	↑ HDL-C [41]	↑ Atherogenic index; ↑ Triglycerides; ↑ Dyslipidemia [41]	—	—
Blood pressure		↑ Hypertension [41]		—
Glucose metabolism	↑ Triglyceride–glucose index [41]		—	—
Adiposity	↑ Central adiposity [41]		—	—
Smoking		↑ Prevalence of smokers [41]	—	—
Metabolic syndrome/risk-factor clustering	—	—	↑ Metabolic syndrome; ↑ Diabetes [48]	
**Subclinical atherosclerosis**				
Carotid atherosclerosis	**↑** Carotid plaque within the high/very-high SCORE category [41]	↑ Carotid IMT ↑ Carotid plaque overall [41]		↑ Subclinical Atherosclerosis [51]
Coronary atherosclerosis	—	—		↑ Vulnerable coronary plaques [53]
Peripheral atherosclerosis disease/multisite disease	—	—		↑ Femoral and multisite disease [52]
**Clinical CV events and mortality**				
Coronary events	—	—		↑ CVD prevalence [55]
CV mortality	—			↑ CV events and CV mortality [56]

↑ indicate a higher or lower value compared with patients of the opposite sex within the same disease. Dashes (—) reflect the absence of sex-disaggregated data rather than negative findings. axSpA, axial spondy-loarthritis; CHD, coronary heart disease; CVD, CV disease; HDL-C, high-density lipoprotein cholesterol; IMT, intima-media thickness; PsA, psoriatic arthritis; SCORE, Systematic Coronary Risk Evaluation; VTE, venous thromboembolism.

**Table 3:** Sex-related differences in inflammatory drivers of CV risk in axial spondyloarthritis and psoriatic arthritis.

	axSpA		PsA	
	Female	Male	Female	Male
Inflammation–lipid link	↑ APR associated with triglycerides [89]	↑ APR associated with HDL-cholesterol [89]	—	—
Inflammation–glucose link	↑ APR associated with insulin-resistance parameters; Independent inflammation–glucose relationship seen only in female patients [89]		—	—
Adiposity–disease activity link	High ASDAS/BASDAI associated with ↑ body-fat percentage and fat mass index [78]		—	—
Residual inflammatory burden	↑ BASDAI [9,10,15]/ASDAS [20]		↑ DASPA [20]	↑ Reduction in plaque progression and vascular inflammation on TNFi [92]

↑ indicate a higher or lower value compared with patients of the opposite sex within the same disease. Dashes (—) reflect the absence of sex-disaggregated data rather than negative findings. Blank cells indicate that no specific finding was reported for that sex. APR, acute-phase reactants; ASDAS, Ankylosing Spondylitis Disease Activity Score; axSpA, axial spondyloarthritis; BASDAI, Bath Ankylosing Spondylitis Disease Activity Index; DASPA, Disease Activity in PSoriatic Arthritis; HDL-C, high-density lipoprotein cholesterol; PsA, psoriatic arthritis; TNFi, tumor necrosis factor inhibitor.

**Table 4:** Summary of current evidence, major limitation and future research priority investigating sex differences in inflammatory burden and CV risk in SpA.

Domain	Current Evidence	Major Limitation	Future Research Priority
Inflammatory burden	Female patients generally report greater disease activity, symptom burden, and poorer treatment persistence despite less radiographic progression in some settings	Heterogeneous disease activity measures; limited mechanistic data	Longitudinal evaluation of inflammatory trajectories powered for sex-stratified analysis
Traditional CV risk factors	Sex differences observed in both axSpA and PsA	Limited large-scale sex-stratified cohorts	Large-scale sex-stratified studies
Subclinical atherosclerosis	Evidence inconsistent in axSpA; male predominance suggested in PsA	Small sample size; heterogeneous imaging approaches	Standardized vascular imaging studies powered for sex-stratified analysis
CV events & mortality	Sex differences remain inconclusive, particularly in axSpA	Few sex-specific analyses	Dedicated sex-stratified outcome studies
Role of Sex hormones/Adipokines/body composition	Biological plausibility supported	Limited prospective data and scarce information on menopausal status, reproductive stage, and hormonal therapy exposure	Hormonal profiling and mechanistic studies evaluating the role of sex hormones in inflammatory burden and cardiovascular risk, particularly across different reproductive stages, menopausal status, and hormonal therapy exposure.

## Data Availability

No new data were created or analyzed in this study. Data sharing is not applicable to this article.

## Abbreviations

The following abbreviations are used in this manuscript:

ACR	American College of Rheumatology
anti-TNF	Anti-tumor necrosis factor
APR	Acute-phase reactants
AS	Ankylosing spondylitis
ASAS40	Assessment of SpondyloArthritis International Society 40% response
ASDAS	Axial Spondyloarthritis Disease Activity Score
AtheSpAin	Atherosclerosis in Spondyloarthritis cohort
axSpA	Axial spondyloarthritis
BASDAI	Bath Ankylosing Spondylitis Disease Activity Index
bDMARDs	Biologic disease-modifying antirheumatic drugs
BMI	Body mass index
BP	Blood pressure
b/tsDMARDs	Biologic and targeted synthetic disease-modifying antirheumatic drugs
CHD	Coronary heart disease
CRP	C-reactive protein
csDMARDs	Conventional synthetic disease-modifying antirheumatic drugs
CV	Cardiovascular
CVD	Cardiovascular disease
DAPSA	Disease Activity Index for Psoriatic Arthritis
DIP	Distal interphalangeal
ESR	Erythrocyte sedimentation rate
HDL-C	High-density lipoprotein cholesterol
HLA-B27	Human leukocyte antigen B27
HR	Hazard ratio
IBD	Inflammatory bowel disease
IBP	Inflammatory back pain
IFNα	Interferon alpha
IL	Interleukin
IL-12/23i	Interleukin-12/23 inhibitor
IL-17i	Interleukin-17 inhibitor
IL-23i	Interleukin-23 inhibitor
IMT	Intima-media thickness
JAKi	Janus kinase inhibitor
MACE	Major adverse cardiovascular events
MASES	Maastricht Ankylosing Spondylitis Enthesitis Score
MDA	Minimal disease activity
MHC	Major histocompatibility complex
MRI	Magnetic resonance imaging
NSAIDs	Non-steroidal anti-inflammatory drugs
nr-axSpA	Non-radiographic axial spondyloarthritis
OR	Odds ratio
PsA	Psoriatic arthritis
PsO	Psoriasis
r-axSpA	Radiographic axial spondyloarthritis
SCORE	Systematic Coronary Risk Evaluation
SIJ	Sacroiliac joint
SpA	Spondyloarthritis
TIA	Transient ischaemic attack
TNFi	Tumor necrosis factor inhibitor
VTE	Venous thromboembolism
